# When Caring Hurts: The Buffering Role of Social Support in Emergency Department Nurses’ Coping With Secondary Traumatic Stress

**DOI:** 10.1155/jonm/8247254

**Published:** 2026-05-26

**Authors:** Ya-Hsin Chen, Yi-Ya Chang, I.-Ting Wang, Shu-Hung Chang

**Affiliations:** ^1^ Department of Gerontology and Health Care Management, Chang Gung University of Science and Technology, Taoyuan City, Taiwan, cgust.edu.tw; ^2^ Department of Nursing, Linkou Chang Gung Memorial Hospital, Taoyuan City, Taiwan, cgmh.org.tw; ^3^ Department of Nursing, Chang Gung University of Science and Technology, Taoyuan City, Taiwan, cgust.edu.tw; ^4^ Department of Health Management, Taipei Chang Gung Memorial Hospital, Taipei, Taiwan, cgmh.org.tw; ^5^ Director of Department of Gerontology and Health Care Management, & Geriatric and Long-Term Care Research Center, Chang Gung University of Science and Technology, Taoyuan City, Taiwan, cgust.edu.tw; ^6^ Department of Gastroenterology and Hepatology, Linkou Chang Gung Memorial Hospital, Taoyuan City, Taiwan, cgmh.org.tw

**Keywords:** coping strategies, emergency department nurses, mediation, nursing management, secondary traumatic stress, social support

## Abstract

**Background:**

Emergency department nurses routinely operate in fast‐paced and emotionally charged environments, where constant exposure to trauma places them at significant risk for developing secondary traumatic stress. Robust coping mechanisms and supportive social networks serve as critical buffers against these psychological effects.

**Aim:**

This study explored how coping strategies and social support interact to influence secondary traumatic stress among emergency department nurses in Taiwan, emphasizing the mediating function of social support.

**Methods:**

A cross‐sectional design was applied between June and August 2024, enrolling 305 emergency department nurses from two tertiary medical centers. Standardized questionnaires were used to measure secondary traumatic stress, coping styles, and perceived support from supervisors, peers, and family. Descriptive and inferential analyses—including independent‐sample *t*‐tests, Pearson correlations, and stepwise multiple regression—were conducted to examine relationships among study variables. Mediation effects were examined with the PROCESS macro (5000 bootstrap resamples).

**Results:**

Almost all respondents (96.1%) experienced at least mild or higher levels of secondary traumatic stress, and 63.3% reported moderate‐to‐severe levels. Avoidant coping emerged as the strongest predictor. Mediation analyses further demonstrated that supervisor support partially mediated the association between avoidant coping and secondary traumatic stress, whereas family support significantly mediated the relationship between emotion‐focused coping and secondary traumatic stress.

**Conclusion:**

Secondary traumatic stress is widespread among emergency department nurses. Avoidant coping heightens susceptibility, whereas supportive supervision and family involvement can mitigate adverse effects.

**Implications:**

Nurse leaders should develop programs that cultivate adaptive coping and enhance both organizational and family‐based support to strengthen resilience and improve care quality.

## 1. Background

In recent years, shifts in healthcare systems, environmental change, emerging infectious diseases, and major crises such as pandemics and natural disasters have caused substantial loss of life and property while placing heavy demands on public health and psychological resilience. The responsibilities of frontline responders—including police officers, firefighters, rescue workers, and clinical staff—become unpredictable under such circumstances, leaving them highly vulnerable to occupational stress [1, 2].

Emergency department nurses, as frontline providers of acute care, routinely manage critically ill patients following traumatic events such as earthquakes, traffic accidents, fires, and violent incidents. In addition to repeated exposure to trauma, they are frequently confronted with rapid patient deterioration and psychological distress, which further compounds their workloads in already strained healthcare environments. Such indirect exposure places emergency department nurses at considerable risk of secondary traumatic stress (STS), a recognized occupational hazard associated with emotional and physiological symptoms such as intrusive recollections, sleep disturbances, somatic symptom disorder, concentration difficulties, anxiety, depression, and fatigue [1, 3–5]. These symptoms can lead to decreased job satisfaction, diminished performance, increased turnover, and workforce instability [2, 6–8]. In Taiwan, emergency department overcrowding—driven by the National Health Insurance system, rising patient inflow, insufficient bed capacity, and high staff turnover—exacerbates occupational stress among emergency department nurses [9].

Although STS is often regarded as a transient, inherent response to caregiving, persistent exposure without adequate mitigation may lead to long‐term psychological sequelae [10]. Nonmodifiable demographic and occupational factors—including gender, years of experience, personality traits, marital status, and work schedule—have been linked to STS. By contrast, modifiable psychosocial factors such as coping strategies and social support are increasingly recognized as crucial to alleviate its impact [7, 11, 12].

Coping strategies—defined as cognitive and behavioral efforts to manage stress—can be categorized as problem‐focused, emotion‐focused, or avoidant [13, 14]. Emergency department nurses often rely on approaches such as avoidance, suppression, help‐seeking, and problem‐solving when facing stress [2]. Evidence consistently indicates that emotion‐focused and avoidant coping are associated with higher levels of STS, whereas problem‐focused coping is linked to lower stress levels [15–17].

Social support—defined as the provision of help, care, and affirmation through interpersonal interactions [18]—is a vital protective factor. Prior research showed that social support alleviates the emotional burden of caregiving and buffers the impact of STS [19–21].

Recent evidence highlighted the high prevalence of STS among emergency department nurses; a systematic review and meta‐analysis revealed a global pooled prevalence of 65%, with an even higher prevalence in Asia, at 74% (95% confidence interval [CI]: 72%–77%) [5]. A mixed‐methods study in Saudi Arabia further emphasized that STS undermines both nurses’ psychological well‐being and the quality of patient care [22]. Overall, the evidence points to the critical role of the interaction between coping strategies and social support in determining how STS develops. Despite growing evidence on the associations between coping strategies, social support, and STS, several important gaps remain in the literature.

First, most existing studies have examined coping strategies and social support as independent predictors of STS, with limited attention to the underlying mechanisms through which these variables interact [12, 16]. In particular, the potential mediating role of different sources of social support has not been sufficiently explored, despite its well‐established buffering function in stress processes, as originally proposed in the stress‐buffering model [23] and supported by recent empirical evidence [20].

Second, the existing evidence is derived from Western contexts, and few empirical studies have been conducted in Asian healthcare settings [5, 7]. Cultural norms may shape coping behaviors, emotional expression, and the utilization of social support, potentially influencing the development and experience of STS [24–26].

Third, research focusing specifically on emergency department nurses who are exposed to high‐intensity and unpredictable traumatic events remains limited, despite their heightened vulnerability to STS [8, 22].

To address these gaps, the present study examines the relationships among coping strategies, social support, and STS among emergency department nurses in Taiwan, with a particular focus on the mediating role of social support.

Accordingly, this study pursued two main objectives (1) to explore the relationships among coping strategies, social support, and STS among emergency department nurses in Taiwan and (2) to assess the mediating function of social support within these associations. The findings are expected to provide nursing managers with evidence‐based insights to implement interventions that promote psychological health, enhance workforce resilience, and ultimately safeguard patient safety.

## 2. Methods

### 2.1. Theoretical Framework

**FIGURE 1:**
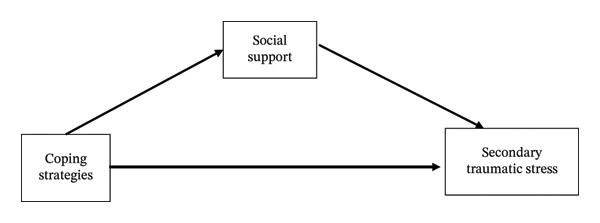
Conceptual framework informed by stress and coping theory and the stress‐buffering model of social support.

Drawing on stress and coping theory [27], this study conceptualized coping strategies as cognitive and behavioral responses to occupational stressors that influence psychological outcomes, including STS. In addition, the proposed framework was informed by the stress‐buffering model of social support [23], which posits that social support mitigates the adverse effects of stress on mental health. Accordingly, the conceptual framework posited coping strategies as the primary independent variables, STS as the outcome variable, and social support as a mediating mechanism through which coping strategies exert their effects (Figure 1).

### 2.2. Study Design and Setting

Employing a cross‐sectional approach and purposive sampling, the study recruited emergency department nurses from two tertiary medical centers—one located in northern Taiwan and one in southern Taiwan. Both institutions are large‐scale teaching hospitals and serve as major regional referral centers, with high annual emergency department visit volumes and comprehensive emergency and trauma care services. These centers manage a broad spectrum of high‐acuity cases and represent key emergency care hubs within their respective regions. Participation was limited to nurses who voluntarily consented to join.

### 2.3. Participants, Sample Size, and Sampling

The required sample size for multiple linear regression was estimated using G ∗ Power 3.1.0 [28], with an effect size of 0.15, *α* = 0.05, power (1‐β) = 0.95, and 11 independent variables. The estimated minimum sample size was 178. Considering a 20% attrition rate, the final required sample size was 214.

Inclusion criteria were as follows: emergency department nurses with more than 3 months of work experience in the emergency department, the ability to use mobile communication software or a computer, and the capacity to complete an online questionnaire. The only exclusion criterion was declining to participate.

### 2.4. Data Collection

The data were gathered between June and August 2024. Recruitment information was disseminated via both online and physical posters within specific hospital units. Participants accessed the study information and consent form via a QR code and completed the Google online questionnaire anonymously. The study ensured voluntary participation, allowing respondents to withdraw at any point without adverse consequences. Data from withdrawn participants were systematically excluded and permanently deleted.

### 2.5. Instruments

#### 2.5.1. Demographics Questionnaire

Demographic data, including gender, age, religious background, and duration of emergency department experience, were collected using a structured questionnaire.

#### 2.5.2. Secondary Traumatic Stress Scale–Chinese Version (STSS‐CV)

Developed in alignment with the DSM‐5 criteria for posttraumatic stress disorder, this instrument measures both the presence and intensity of STS symptoms. The Chinese translation was provided by He et al. [29], and the scale has been widely used in studies involving healthcare professionals and clinical nurses. It comprises two subscales: stress and intrusion and avoidance. Responses were rated on a five‐point Likert scale (1–5), with higher scores representing greater frequency of STS symptoms. The severity of STS was classified into five levels: scores under 28 denoted the absence of symptoms; 28–37 indicated mild, 38–43 moderate, 44–48 high, and 49 or above represented severe stress. In the present study, the STSS‐CV demonstrated good internal consistency (Cronbach’s *α* = 0.88). Although prior validation studies have reported a two‐factor structure [29], exploratory factor analysis in the present sample supported a three‐factor solution reflecting intrusion and hyperarousal, emotional numbing, and avoidance‐related symptoms. Given the exploratory nature of this analysis, the total score was used in subsequent analyses.

#### 2.5.3. Brief Coping Orientation to Problems Experienced (Brief COPE) Scale

The Brief COPE scale, originally developed by Carver [30], was adopted in this study. This instrument contains 28 items grouped into three domains: “emotion‐focused coping,” “problem‐focused coping,” and “avoidant coping.” Each item was rated on a four‐point Likert scale from 1 *(‘rarely used’)* to 4 *(‘frequently used’)*, with higher ratings indicating a greater inclination toward the respective coping strategy. In the present study, the Brief COPE demonstrated good internal consistency, with a Cronbach’s *α* coefficient of 0.91 for the overall scale. The reliability coefficients for the subscales were *α* = 0.79 for emotion‐focused coping, *α* = 0.80 for problem‐focused coping, and *α* = 0.84 for avoidant coping. Consistent with prior research, the Brief COPE has been conceptualized using higher order groupings, most commonly classified into problem‐focused coping, emotion‐focused coping, and avoidant coping, to facilitate theoretical interpretation and comparability across studies. Exploratory factor analysis using principal component analysis in the present sample suggested a multidimensional structure, with several components exceeding eigenvalues of 1, indicating potential variability in factor structure across populations and contexts. Therefore, in line with established literature, the three‐domain classification was retained for subsequent analyses rather than adopting a sample‐specific factor solution.

#### 2.5.4. Social Support Scale

The Social Support Scale, developed by Wong et al. [31], was also used in this study. Comprising 12 items, the instrument measures three aspects of support—supervisory, collegial, and familial. Responses are rated on a five‐point Likert scale, with higher values reflecting greater perceived support.

Social support was assessed using a multidimensional social support scale encompassing supervisor support, peer support, and family support. In the present sample of Taiwanese emergency department nurses, the overall scale demonstrated good internal consistency (Cronbach’s *α* = 0.871). The subscales also showed excellent reliability, with Cronbach’s *α* values of 0.869 for supervisor support, 0.881 for peer support, and 0.887 for family support.

Exploratory factor analysis supported a three‐factor structure corresponding to these theoretically defined domains, indicating adequate construct validity of the scale in this population. These findings are consistent with prior applications of the scale in Taiwanese nursing samples and support its suitability for assessing perceived social support among emergency department nurses.

### 2.6. Ethical Considerations

Ethical clearance for this research was granted by the Institutional Review Board of the participating medical center (IRB No. 202400413B0). All participants were provided with detailed study information and an electronic informed consent form prior to participation. Informed consent was obtained online before participants accessed the questionnaire, and participation was entirely voluntary. Participants were informed of their right to withdraw at any time without penalty, and all responses were collected anonymously.

### 2.7. Data Analysis

Statistical computations and analyses were conducted through IBM SPSS Statistics (Version 26.0; IBM Corp., Armonk, NY, USA). Descriptive analyses were conducted to summarize frequencies, percentages, means, and standard deviations to characterize participants’ demographics and study variables. Independent‐samples *t*‐tests were applied to compare STS levels across demographic subgroups, whereas Pearson’s correlations examined relationships among continuous measures. In the final phase of the analysis, a stepwise multiple regression approach was employed to determine the variables exerting the strongest influence on STS. Subsequently, the PROCESS macro in SPSS (Model 4; [32]) with 5000 bootstrap samples was utilized to examine whether social support mediated the association between coping strategies and STS.

In the stepwise multiple regression analysis, changes in regression coefficients across models were interpreted as reflecting shared variance among predictors rather than directional instability. Variables that did not demonstrate sufficient independent explanatory power after accounting for multicollinearity were excluded by the stepwise procedure. This approach was adopted as an exploratory method to identify the most salient predictors of STS and to derive a parsimonious model, given the presence of multiple correlated coping and social support variables and the absence of a priori evidence supporting a fixed variable entry order.

Mediation analyses were conducted using the PROCESS macro, which applies an ordinary least squares regression–based approach with bootstrap resampling to estimate indirect effects.

Unlike structural equation modeling (SEM), this approach does not involve the estimation of a global model; therefore, overall model fit indices (e.g., RMSEA, CFI, TLI) are not applicable. Instead, the adequacy of the mediation model was evaluated based on the significance and magnitude of path coefficients, as well as bias‐corrected bootstrap confidence intervals for indirect effects. The mediation effect was considered statistically significant when the 95% confidence interval did not include zero, in accordance with established methodological recommendations [32].

## 3. Results

### 3.1. Descriptive Statistics

Altogether, data from 305 emergency department nurses were analyzed. Females accounted for 85.9% of the sample, and their mean age was 32.44 years (SD = 7.66), with a mean of 9.35 years (SD = 7.10) of emergency department experience.

Most respondents held a bachelor’s degree or higher (91.5%), were unmarried (63.6%), and reported having no children (68.9%). More than half (58.4%) indicated a religious affiliation (Table 1).

**TABLE 1:** Descriptive statistics for demographic variables, social support, coping strategies, and STS.

Variable	*n* (%)	Variable	Mean ± SD
Gender		Age	32.44 ± 7.66
Male	43 (14.1)	Years of emergency experience	9.35 ± 7.10
Female	262 (85.9)	Social Support Scale	43.17 ± 6.29
Religious beliefs		Supervisor support	12.64 ± 2.96
None	127 (41.6)	Colleague support	14.73 ± 2.63
Religious	178 (58.4)	Family support	15.80 ± 2.77
Marital status		Coping Strategies Scale	57.78 ± 11.94
Married	111 (36.4)	Emotion‐focused coping	21.70 ± 4.58
Single	194 (63.6)	Problem‐focused coping	13.92 ± 3.37
Education level		Avoidant coping	22.16 ± 5.24
Diploma	26 (8.5)	STS score	40.94 ± 9.17
Bachelor or above	279 (91.5)		
Number of children			
None	210 (68.9)		
With children	95 (31.1)		
STS classification			
None (< 28)	12 (3.9)		
Mild (28–37)	100 (32.8)		
Moderate (38–43)	90 (29.5)		
High (44–48)	50 (16.4)		
Severe (> 49)	53 (17.4)		

Abbreviations: SD, standard deviation; STS, secondary traumatic stress.

Participants demonstrated a mean score of 43.17 (SD = 0.36) on the Social Support Scale, indicating a moderately high perception of available support. Family support showed the highest average rating, followed by support from colleagues and supervisors. On the Coping Strategies Scale, the overall mean was 57.78 (SD = 0.68), with avoidant coping being the predominant strategy, ahead of emotion‐focused and problem‐focused coping.

With regard to STS, only 3.9% of participants reported having no symptoms. Nearly all respondents (96.1%) experienced at least mild symptoms, and 63.3% reported moderate‐to‐severe levels (Table 1).

### 3.2. Group Comparisons

Results from the independent‐samples *t*‐tests showed no significant variation in STS among the demographic groups examined (Table 2).

**TABLE 2:** Differences in STS across demographic variables (*N* = 305).

	**Mean ± SD**	***t***	**p**

Gender		−1.68	0.093
Male	38.77 ± 9.37		
Female	41.30 ± 9.10		
Religious beliefs		−0.80	0.422
None	40.44 ± 9.57		
Religious	41.30 ± 8.88		
Marital status		0.69	0.489
Single	41.22 ± 9.73		
Married	40.46 ± 8.20		
Education level		−1.63	0.105
Diploma	38.15 ± 8.27		
Bachelor or above	41.20 ± 9.21		
Number of children		0.23	0.815
None	41.02 ± 9.87		
With children	40.76 ± 7.42		

Abbreviations: SD, standard deviation; STS, secondary traumatic stress.

### 3.3. Correlation Analysis

No statistically significant relationship was found between STS and participants’ age or length of experience in emergency department nursing.

However, supervisor support (*r* = −0.29, *p* < 0.001), colleague support (*r* = −0.20, *p* < 0.001), and family support (*r* = −0.21, *p* < 0.001) were inversely related to STS. Conversely, emotion‐focused coping (*r* = 0.27, *p* < 0.001), problem‐focused coping (*r* = 0.39, *p* < 0.001), and avoidant coping (*r* = 0.60, *p* < 0.001) demonstrated positive associations with it (Table 3).

**TABLE 3:** Pearson’s correlation analysis among secondary traumatic stress, coping strategies, and social support variables (*N* = 305).

	**STS**	**Age**	**Years of emergency experience**	**Supervisor support**	**Colleague support**	**Family support**	**Emotion-focused coping**	**Problem-focused coping**	**Avoidant coping**

STS	1	< 0.01	0.01	−0.29^∗∗∗^	−0.20^∗∗∗^	−0.21^∗∗∗^	0.27^∗∗∗^	0.39^∗∗∗^	0.60^∗∗∗^

Abbreviation: STS, secondary traumatic stress.

^∗∗∗^ *p* < 0.001.

### 3.4. Factors Associated With STS

Stepwise regression revealed that avoidant coping exerted the greatest influence on STS, explaining 36% of the variance. When emotion‐focused coping, supervisor support, and family support were included, the explained variance increased to 42% (*R* ^2^ = 0.43, adjusted *R* ^2^ = 0.42, *F* = 55.82, *p* < 0.001) (Table 4).

**TABLE 4:** Stepwise regression of factors associated with STS among emergency department nurses (*N* = 305).

Model		*B*	SE	*β*	*t* value	*R* ^2^	Adjusted *R* ^2^
1	Avoidant coping	1.06	0.08	0.60	13.20^∗∗∗^	0.37	0.36

2	Avoidant coping	1.32	0.10	0.76	12.74^∗∗∗^	0.40	0.39
	Emotion‐focused coping	−0.46	0.12	−0.23	−3.89^∗∗∗^		

3	Avoidant coping	1.25	0.10	0.71	11.92^∗∗∗^	0.42	0.41
	Emotion‐focused coping	−0.42	0.12	−0.21	−3.59^∗∗∗^		
	Supervisor support	−0.48	0.14	−0.16	−3.46^∗∗^		

4	Avoidant coping	1.20	0.11	0.69	11.29^∗∗∗^	0.43	0.42
	Emotion‐focused coping	−0.35	0.12	−0.17	−2.85^∗∗^		
	Supervisor support	−0.42	0.14	−0.14	−2.96^∗∗^		
	Family support	−0.33	0.16	−0.10	−2.09^∗^		

Abbreviation: STS, secondary traumatic stress.

^∗^ *p* < 0.05.

^∗∗^ *p* < 0.01.

^∗∗∗^ *p* < 0.001.

### 3.5. Mediation Analysis

**FIGURE 2:**
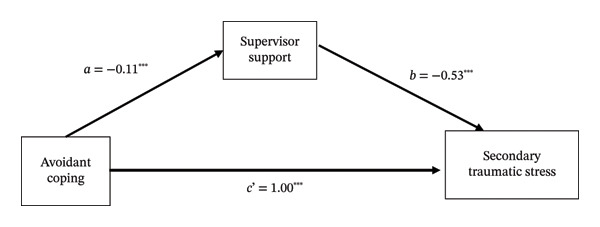
Mediation model of supervisor support in the relationship between avoidant coping and secondary traumatic stress among emergency department nurses. Note: All coefficients are significant at *p* < 0.001.

**FIGURE 3:**
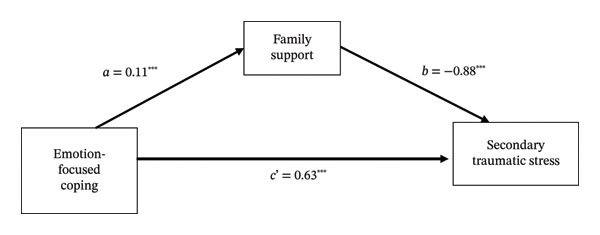
Mediation model of family support in the relationship between emotion‐focused coping and secondary traumatic stress among emergency department nurses. Note: All coefficients are significant at *p* < 0.001.

PROCESS mediation analysis identified two significant indirect pathways. Supervisor support partially mediated the relationship between avoidant coping and STS (see Table 5 and Figure 2). Similarly, family support partially mediated the association between emotion‐focused coping and STS (see Table 6 and Figure 3).

**TABLE 5:** Mediation analysis of supervisor support in the relationship between avoidant coping and secondary traumatic stress among emergency department nurses (*N* = 305).

Effect	Path	*β*	95% CI	SE	*t*	*p*
Direct effect	Avoidant coping ⟶ Secondary traumatic stress	1.00 (c’)	0.84, 1.15	0.08	12.47	< 0.001

Indirect effect	Avoidant coping ⟶ Supervisor Support	−0.11 (a)	−0.17, −0.05	0.03	−3.52	< 0.001
	Supervisor Support ⟶ Secondary traumatic stress	−0.53 (b)	−0.81, −0.25	0.14	−3.77	< 0.001

Total effect	Avoidant coping ⟶ Secondary traumatic stress	1.06	0.90, 1.22	0.08	13.20	< 0.001

*Note:* Analyses were adjusted for covariates including age, gender, religious beliefs, marital status, education level, number of children, and years of emergency department experience.

**TABLE 6:** Mediation analysis of family support in the relationship between emotion‐focused coping and secondary traumatic stress among emergency department nurses (*N* = 305).

Effect	Path	*β*	95% CI	SE	*t*	*p*
Direct effect	Emotion‐focused coping ⟶ Secondary traumatic stress	0.63 (c’)	0.42, 0.84	0.11	5.79	< 0.001

Indirect effect	Emotion‐focused coping ⟶ Family support	0.11 (a)	0.05, 0.18	0.03	3.32	< 0.001
	Family support ⟶ Secondary traumatic stress	−0.88 (b)	−1.23, −0.52	0.18	−4.88	< 0.001

Total effect	Emotion‐focused coping ⟶ Secondary traumatic stress	0.53	0.31, 0.75	0.11	4.78	< 0.001

*Note:* Analyses were adjusted for covariates including age, gender, religious beliefs, marital status, education level, number of children, and years of emergency department experience.

**FIGURE 4:**
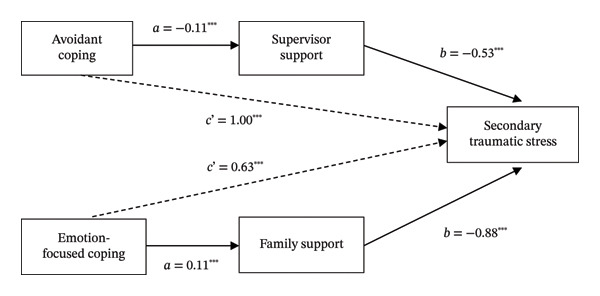
Integrated model summarizing direct and indirect effects of coping strategies on STS via social support. Note: Dashed lines indicate direct effects; solid lines indicate mediated pathways. Only statistically significant paths are shown; ^∗∗∗^ *p* < 0.001.

To further enhance the clarity of relationships among variables, key associations identified in the correlation and regression analyses are visually summarized in Figure 4, allowing readers to understand the relationships more clearly among variables as well as the direction of both direct and indirect effects.

## 4. Discussion

The present study confirmed that STS is highly prevalent among emergency department nurses in Taiwan, reflecting a considerable psychological burden within this workforce. Nearly all participants reported at least mild symptoms, and almost two‐thirds (63.3%) experienced moderate‐to‐severe levels.

Findings of this study align with prior research indicating that avoidant coping is a key factor linked to STS, followed by emotion‐focused coping, while supervisor and family support functioned as protective factors [33–35]. The mediation analysis further revealed two significant indirect pathways: supervisor support partially mediated the relationship between avoidant coping and STS, whereas family support mediated the link between emotion‐focused coping and STS. Collectively, these results underscore the intertwined effects of coping mechanisms and social support on nurses’ psychological well‐being, reinforcing the importance of multitiered intervention approaches [12, 36].

The prevalence of moderate‐to‐severe STS among Taiwanese emergency department nurses (63.3%) was comparable to international estimates (52%–65%) [8, 36, 37]. However, cultural norms within Confucian‐influenced societies may influence how nurses interpret and express psychological distress. In such contexts—where moral duty, filial piety, and social harmony are emphasized—nurses may be more inclined to suppress emotional disclosure to maintain professional composure and avoid burdening others [38]. Consequently, the true prevalence of severe STS in Taiwan may be underestimated [5, 29]. These interpretations should be viewed as context‐informed hypotheses rather than definitive conclusions, as cultural values were not directly measured in this study. From a cross‐cultural perspective, differences in the perception and utilization of social support and coping strategies have been increasingly documented in recent research. Cultural context plays a critical role in shaping how individuals perceive, process, and utilize social support in response to stress [24–26, 39, 40]. In Western settings, individuals are more likely to engage in open emotional expression and actively seek professional or peer support when experiencing psychological distress. In contrast, in East Asian cultures influenced by collectivist and Confucian values, emotional restraint, role obligation, and the maintenance of social harmony may shape how individuals perceive stress and utilize support systems. These patterns are grounded in foundational cultural frameworks [24, 25] and are further supported by recent cross‐cultural research [40]. Such cultural differences may influence not only the expression of STS but also the relative salience and effectiveness of different sources of social support. Recent evidence suggests that collective or relational forms of coping are more prominent in collectivist cultural contexts, where individuals rely more on family‐ and group‐based resources to manage stress [26, 40]. This perspective may partially explain the stronger role of family support observed in the present study.

However, these cross‐cultural interpretations remain speculative, as cultural variables were not directly assessed. Future studies should incorporate cross‐cultural or comparative designs to further examine how cultural contexts shape coping processes and social support mechanisms in relation to STS.

Although coping strategies are commonly categorized as problem‐focused, emotion‐focused, and avoidant, the present findings indicated that only emotion‐focused and avoidant coping were independently associated with STS. The uncontrollable and unpredictable nature of traumatic events in emergency care likely limits the effectiveness of problem‐focused coping in alleviating psychological distress. In contrast, emotion‐focused and avoidant coping—though potentially providing short‐term relief—may hinder emotional processing and intensify distress over time, increasing vulnerability to STS [16, 17].

Recent studies likewise report that avoidant and maladaptive emotion‐focused coping are linked to higher levels of STS across helping professions, including among social workers and refugee service providers [12, 35, 41, 42].

Although problem‐focused coping is often considered adaptive, its positive bivariate correlation with STS in the present study may reflect the high‐acuity and largely uncontrollable nature of emergency care. In such contexts, greater reliance on problem‐focused coping may indicate higher exposure to traumatic events and increased professional responsibility rather than effective stress resolution.

Importantly, however, this association was observed only at the bivariate level and did not remain significant in multivariable analyses, further indicating that problem‐focused coping did not exert an independent effect on STS after accounting for other coping strategies and social support.

The findings underscore the mediating functions of supervisor and family support in linking coping strategies with STS. Although peer support showed a negative bivariate correlation with STS, it did not remain significant in multivariable analyses, which may reflect shared variance with supervisor and family support and reduced unique explanatory power when modeled simultaneously.

Supervisor support buffered the detrimental effects of avoidant coping, emphasizing the protective role of trauma‐informed leadership. Supervisors who maintain open communication, acknowledge staff efforts, and provide emotional guidance may help nurses reframe avoidant responses and mitigate long‐term consequences [43]. Meanwhile, the mediating role of family support indicates that within supportive family environments, emotional expression may shift from a vulnerability to a protective factor. Families that offer empathy, validation, and reassurance can help transform emotion‐focused coping from maladaptive to adaptive [12, 39, 44].

These insights suggest that interventions should simultaneously strengthen supervisory resources and incorporate family‐based psychoeducation to foster resilience. Avoidant coping, though common in uncontrollable stress situations, is associated with intrusive thoughts, sleep disturbances, and fatigue, thereby exacerbating STS over time [16, 45]. Evidence‐based interventions—such as mindfulness training, resilience‐building, and structured debriefings—have demonstrated efficacy in mitigating secondary trauma [46, 47]. Incorporating brief practices into daily workflows, such as mindfulness during shift transitions, may normalize adaptive coping and reduce reliance on avoidance [5, 48].

The protective role of supervisor support identified in this study echoes previous evidence linking supportive leadership with reduced burnout and greater psychological safety [12, 44]. In Taiwan, where nurse turnover remains a persistent challenge, developing leadership training programs emphasizing trauma‐informed and empathetic management is crucial. Furthermore, the mediating role of family support highlights the need for hospitals to engage families through psychoeducational initiatives. Family involvement—often neglected in occupational health frameworks—may constitute a critical informal support system that buffers emergency department nurses from psychological harm.

Beyond individual and interpersonal domains, systemic stressors such as overcrowding, excessive workloads, and staffing shortages further exacerbate STS. Organizational reforms—including optimizing patient flow, maintaining safe nurse–patient ratios, and implementing retention incentives—are essential to alleviate these structural burdens [9, 22]. In parallel, resilience training, peer‐support groups, and employee assistance programs have shown effectiveness in improving psychological outcomes [5]. Hospitals should integrate adaptive coping training, mindfulness, and acceptance and commitment therapy into professional development while implementing family‐inclusive support programs to strengthen the psychosocial protection of healthcare staff.

## 5. Implications for Nursing Management

These findings yield several important implications for nursing leadership, highlighting strategic, educational, and organizational priorities to enhance workforce resilience.

First, systematic monitoring of STS among emergency department nurses should be established through the routine use of validated screening tools to enable early identification and timely intervention for at‐risk staff. Early recognition facilitates access to psychosocial support and helps prevent progression to severe psychological distress.

Second, professional development programs should include training aimed at reducing reliance on avoidant and maladaptive emotion‐focused coping. Evidence‐based interventions—such as mindfulness‐based stress reduction, resilience‐building, and acceptance and commitment therapy—have demonstrated efficacy in mitigating secondary trauma and strengthening coping capacity [5, 41, 48]. Embedding these practices into daily workflows may help normalize adaptive coping in high‐stress environments.

Third, leadership development must be prioritized to enhance supervisory support. Administrators should equip nurse leaders with trauma‐informed competencies, including empathetic communication, stress recognition, and emotional intelligence. Effective supervisory support not only directly reduces STS but also buffers nurses from the negative impact of avoidant coping [43].

Fourth, hospitals should adopt family‐inclusive strategies. Psychoeducational workshops for family members can increase understanding of the emotional demands of emergency department nursing and enable families to provide consistent and empathetic support. Strengthening family involvement may amplify the protective effects of emotion‐focused coping, as identified in this study [39].

Finally, organizational and policy‐level reforms are essential to address systemic stressors such as overcrowding, excessive workloads, and staffing shortages. Measures including patient‐flow optimization, maintenance of safe nurse–patient ratios, and implementation of retention incentives should be integrated with individual‐, supervisory‐, and family‐level interventions to create a comprehensive, multilayered system of protection against STS [9, 22].

Taken together, from a nursing management perspective, these findings underscore the importance of organizational policies that promote psychological safety and trauma‐informed supervision. Structured supervisory models that emphasize emotional validation, open communication, and access to support resources may help mitigate the impact of STS. At the policy level, integrating mental health support into routine occupational health programs and establishing clear referral pathways may further strengthen institutional support for emergency department nurses.

## 6. Limitations and Future Research Directions

This study has several limitations that should be considered when interpreting its findings.

First, the cross‐sectional design allows identification of associations among variables but precludes any inference of causality. Although mediation analysis was conducted, this design does not allow the establishment of temporal ordering among coping strategies, social support, and STS. Accordingly, the mediation findings should be interpreted as statistical associations rather than evidence of causal pathways. Therefore, longitudinal or experimental studies are needed to establish temporal relationships and evaluate the effectiveness of interventions—such as enhanced social support or coping‐skills training—in reducing STS. Such approaches would provide stronger causal evidence to better support the psychological well‐being of emergency department nurses.

Second, data were collected exclusively from emergency departments in two tertiary medical centers in Taiwan. Although these hospitals are major urban referral institutions, the findings may not generalize to nurses working in different settings such as community hospitals, rural facilities, or trauma‐specialized centers. Given that these are high‐volume tertiary emergency departments, nurses may be exposed to more frequent high‐acuity and traumatic cases, as well as greater organizational demands, which may have influenced the observed levels of STS and coping patterns. In addition, the use of purposive and voluntary sampling may have introduced selection bias, as nurses experiencing higher levels of stress or a greater interest in psychosocial issues might have been more inclined to participate. Replication across diverse clinical settings, geographic regions, and healthcare systems, using probability‐based or multicenter sampling strategies, is recommended to improve external validity and generalizability.

Third, self‐reported measures inherently carry the risk of response and recall biases, given that participants may distort their answers, consciously or otherwise, to present themselves in a more favorable manner.

In addition, participants’ pre‐existing mental health conditions, such as anxiety or depression, were not assessed or used as exclusion criteria. As these conditions may influence the experience and reporting of STS, their potential confounding effects cannot be ruled out. Future studies should therefore consider controlling for baseline mental health status or incorporating clinical screening measures.

Future research should employ mixed‐methods approaches, combining qualitative interviews, supervisor evaluations, and objective physiological indicators of stress to enhance methodological robustness.

Finally, while this study focused on coping strategies and social support, other organizational and contextual factors—such as staffing ratios, shift schedules, managerial climate, and cumulative trauma exposure—were not examined. Future investigations should adopt an ecological or systems‐level perspective that integrates individual‐, organizational‐, and policy‐level determinants to capture the multifaceted nature of STS in emergency department nursing.

## 7. Conclusion

This study demonstrated that STS is highly prevalent among emergency department nurses in Taiwan, with most participants reporting at least mild symptoms and nearly two‐thirds experiencing moderate‐to‐severe levels. Both avoidant and emotion‐focused coping were significant predictors of STS, while supervisor and family support served as mediating buffers mitigating their adverse effects. Collectively, these findings highlight the interdependent relationship between coping mechanisms and social support in sustaining nurses’ psychological well‐being and professional functioning.

For nursing management, these results underscore the need for multilevel, integrated interventions—including the promotion of adaptive coping strategies, the development of trauma‐informed and empathetic leadership, the engagement of families in supportive roles, and the implementation of organizational reforms. By adopting such a comprehensive approach, healthcare organizations can strengthen the emotional resilience and retention of emergency department nurses, enhance psychological safety, and foster an environment conducive to the consistent delivery of compassionate, high‐quality patient care.

## Funding

This work was supported by the Chang Gung Medical Research Fund (CMRPG3P0421).

## Disclosure

The funding agency had no role in the design, data collection, analysis, interpretation, or publication of this research.

## Ethics Statement

Ethical approval was secured from the Institutional Review Board of Linkou Chang Gung Memorial Hospital (IRB No. 202400413B0), and the investigation conformed to the standards set forth in the Declaration of Helsinki and its later amendments.

## Conflicts of Interest

The authors declare no conflicts of interest.

## Data Availability

The data that support the findings of this study are available from the corresponding author upon reasonable request.

## References

[1] Greinacher A. , Derezza-Greeven C. , Herzog W. , and Nikendei C. , Secondary Traumatization in First Responders: A Systematic Review, European Journal of Psychotraumatology. (2019) 10, no. 1, 10.1080/20008198.2018.1562840. PMC6346705 30719236

[2] Kelly L. , Burnout, Compassion Fatigue, and Secondary Trauma in Nurses: Recognizing the Occupational Phenomenon and Personal Consequences of Caregiving, Critical Care Nursing Quarterly. (2020) 43, no. 1, 73–80, 10.1097/CNQ.0000000000000293. 31789880

[3] Ariapooran S. , Ahadi B. , and Khezeli M. , Depression, Anxiety, and Suicidal Ideation in Nurses With and Without Symptoms of Secondary Traumatic Stress During the COVID-19 Outbreak, Archives of Psychiatric Nursing. (2022) 37, 76–81, 10.1016/j.apnu.2021.05.005. 35337442 PMC8938317

[4] Robinson L. K. , Sterling L. , Jackson J. et al., A Secondary Traumatic Stress Reduction Program in Emergency Room Nurses, SAGE Open Nursing. (2022) 8, 10.1177/23779608221094530. PMC9096200 35574270

[5] Xu Z. , Zhao B. , Zhang Z. et al., Prevalence and Associated Factors of Secondary Traumatic Stress in Emergency Nurses: A Systematic Review and Meta-Analysis, European Journal of Psychotraumatology. (2024) 15, no. 1, 10.1080/20008066.2024.2321761. PMC10911249 38426665

[6] Bock C. , Heitland I. , Zimmermann T. , Winter L. , and Kahl K. G. , Secondary Traumatic Stress, Mental State, and Work Ability in nurses-Results of a Psychological Risk Assessment at a University Hospital, Frontiers in Psychiatry. (2020) 11, 10.3389/fpsyt.2020.00298. PMC7197484 32395109

[7] Lopez J. , Bindler R. J. , and Lee J. , Cross-Sectional Analysis of Burnout, Secondary Traumatic Stress, and Compassion Satisfaction Among Emergency Nurses in Southern California Working Through the COVID-19 Pandemic, Journal of Emergency Nursing. (2022) 48, no. 4, 366–375. e362, 10.1016/j.jen.2022.03.008. 35690484 PMC8958096

[8] Ratrout H. F. and Hamdan‐Mansour A. M. , Secondary Traumatic Stress Among Emergency Nurses: Prevalence, Predictors, and Consequences, International Journal of Nursing Practice. (2020) 26, no. 1, 10.1111/ijn.12767, 2-s2.0-85069835295. 31328356

[9] Lin P. Y. , Kaplan W. , Lin C. H. , and Lee Y. H. , Taiwan’s National Health Insurance at the Emergency Department Following the COVID‐19 Outbreak, Public Health Nursing. (2023) 40, no. 4, 517–527, 10.1111/phn.13186. 36882994

[10] Bride B. E. , Prevalence of Secondary Traumatic Stress Among Social Workers, Social Work. (2007) 52, no. 1, 63–70, 10.1093/sw/52.1.63, 2-s2.0-33947599932. 17388084

[11] Barleycorn D. , Awareness of Secondary Traumatic Stress in Emergency Nursing, Emergency Nurse. (2019) 27, no. 5, 19–22, 10.7748/en.2019.e1957, 2-s2.0-85071773937. 31475501

[12] Kim S. J. and Yeo J. H. , Factors Affecting Posttraumatic Stress Disorder in South Korean Trauma Nurses, Journal of Trauma Nursing. (2020) 27, no. 1, 50–57, 10.1097/JTN.0000000000000482. 31895320

[13] Folkman S. and Moskowitz J. T. , Stress, Positive Emotion, and Coping, Current Directions in Psychological Science. (2000) 9, no. 4, 115–118, 10.1111/1467-8721.00073, 2-s2.0-0034348706.

[14] Wilson G. S. , Pritchard M. E. , and Revalee B. , Individual Differences in Adolescent Health Symptoms: The Effects of Gender and Coping, Journal of Adolescence. (2005) 28, no. 3, 369–379, 10.1016/j.adolescence.2004.08.004, 2-s2.0-19944380813. 15925688

[15] Hamama-Raz Y. and Minerbi R. , Coping Strategies in Secondary Traumatization and Post-traumatic Growth Among Nurses Working in a Medical Rehabilitation Hospital: A Pilot Study, International Archives of Occupational and Environmental Health. (2019) 92, no. 1, 93–100, 10.1007/s00420-018-1354-z, 2-s2.0-85053434489. 30206702

[16] Meyerson J. , Gelkopf M. , Eli I. , and Uziel N. , Stress Coping Strategies, Burnout, Secondary Traumatic Stress, and Compassion Satisfaction Amongst Israeli Dentists: A Cross-Sectional Study, International Dental Journal. (2022) 72, no. 4, 476–483, 10.1016/j.identj.2021.09.006. 34785064 PMC9381368

[17] Rodríguez-Rey R. , Palacios A. , Alonso-Tapia J. et al., Burnout and Posttraumatic Stress in Paediatric Critical Care Personnel: Prediction From Resilience and Coping Styles, Australian Critical Care. (2019) 32, no. 1, 46–53, 10.1016/j.aucc.2018.02.003, 2-s2.0-85044504388. 29605169

[18] Kahn R. L. , Riley M. W. , Aging and Social Support, Aging From Birth to Death: Interdisciplinary Perspectives, 1979, Westview Press, Boulder, Colorado, 77–91.

[19] Alnazly E. , Khraisat O. M. , Al-Bashaireh A. M. , and Bryant C. L. , Anxiety, Depression, Stress, Fear and Social Support During COVID-19 Pandemic Among Jordanian Healthcare Workers, PLoS One. (2021) 16, no. 3, 10.1371/journal.pone.0247679. PMC7954309 33711026

[20] Gurowiec P. J. , Ogińska-Bulik N. , Michalska P. , and Kędra E. , The Relationship Between Social Support and Secondary Posttraumatic Growth Among Health Care Providers Working With Trauma Victims—The Mediating Role of Cognitive Processing, International Journal of Environmental Research and Public Health. (2022) 19, no. 9, 10.3390/ijerph19094985. PMC9104597 35564379

[21] Schug C. , Morawa E. , Geiser F. et al., Social Support and Optimism as Protective Factors for Mental Health Among 7765 Healthcare Workers in Germany During the COVID-19 Pandemic: Results of the VOICE Study, International Journal of Environmental Research and Public Health. (2021) 18, no. 7, 10.3390/ijerph18073827. PMC8038794 33917493

[22] Alshammari B. , Alanazi N. F. , Kreedi F. et al., Exposure to Secondary Traumatic Stress and Its Related Factors Among Emergency Nurses in Saudi Arabia: A Mixed Method Study, BMC Nursing. (2024) 23, no. 1, 10.1186/s12912-024-02018-4. PMC11102619 38762742

[23] Cohen S. and Wills T. A. , Stress, Social Support, and the Buffering Hypothesis, Psychological Bulletin. (1985) 98, no. 2, 310–357, 10.1037/0033-2909.98.2.310, 2-s2.0-0022115624. 3901065

[24] Hofstede G. , Culture’s Consequences: Comparing Values, Behaviors, Institutions, and Organizations Across Nations, 2001, 2nd edition, Sage, Thousand Oaks, CA.

[25] Markus H. R. and Kitayama S. , Culture and the Self: Implications for Cognition, Emotion, and Motivation, Psychological Review. (1991) 98, no. 2, 224–253, 10.1037/0033-295X.98.2.224, 2-s2.0-12044258070.

[26] Szkody E. , Spence A. , Özdoğru A. et al., Social Support and Help-Seeking Worldwide, Current Psychology. (2024) 43, no. 22, 20165–20181, 10.1007/s12144-024-05764-5.

[27] Lazarus R. and Folkman S. , Stress, Appraisal, and Coping, 1984, Springer, New York, NY.

[28] Faul F. , Erdfelder E. , Buchner A. , and Lang A. G. , Statistical Power Analyses Using G∗Power 3.1: Tests for Correlation and Regression Analyses, Behavior Research Methods. (2009) 41, no. 4, 1149–1160, 10.3758/BRM.41.4.1149, 2-s2.0-74949117960. 19897823

[29] He Y. , Liu Z. , Zhang J. , Yao J. , Xiao H. , and Wan H. , Validity and Reliability of the Secondary Traumatic Stress Scale-Chinese Version, Frontiers in Surgery. (2022) 9, 10.3389/fsurg.2022.882712. PMC9010506 35433816

[30] Carver C. S. , You Want to Measure Coping but Your Protocol’s Too Long: Consider the Brief COPE, International Journal of Behavioral Medicine. (1997) 4, no. 1, 92–100, 10.1207/s15327558ijbm0401_6, 2-s2.0-0002255373. 16250744

[31] Wong Y. H. , Lin J. H. , and Liu S. H. , Coping With Work-Nonwork Conflict and Promoting Life Quality of Frontline Employees via Social Support, Journal of Management & Systems. (2008) 15, no. 3, 355–376, https://ir.lib.nycu.edu.tw/bitstream/11536/108021/1/10239863-01503-99.pdf, (In Chinese).

[32] Hayes A. F. , Introduction to Mediation, Moderation, and Conditional Process Analysis: A Regression-Based Approach, 2017, Guilford Publications.

[33] Al Barmawi M. , Shahrouri B. E. , Al Hadid L. et al., Measuring the Prevalence, Warning Signs, and Preventive Measures of Secondary Traumatic Stress Among Critical Care Nurses, BMC Psychiatry. (2025) 25, no. 1, 10.1186/s12888-025-06840-1. PMC12054241 40325395

[34] Piras I. , Usai V. , Contu P. , and Galletta M. , Vicarious Trauma, Coping Strategies and Nurses’ Health Outcomes: An Exploratory Study, AIMS Public Health. (2024) 11, no. 4, 1071–1081, 10.3934/publichealth.2024055. 39802564 PMC11717540

[35] Tsouvelas G. , Kalaitzaki A. , Tamiolaki A. , Rovithis M. , and Konstantakopoulos G. , Secondary Traumatic Stress and Dissociative Coping Strategies in Nurses During the COVID-19 Pandemic: The Protective Role of Resilience, Archives of Psychiatric Nursing. (2022) 41, 264–270, 10.1016/j.apnu.2022.08.010. 36428058 PMC9428110

[36] Woo M. J. and Kim D. H. , Factors Associated With Secondary Traumatic Stress Among Nurses in Regional Trauma Centers in South Korea: A Descriptive Correlational Study, Journal of Emergency Nursing. (2021) 47, no. 3, 400–411, 10.1016/j.jen.2020.08.006. 33229000

[37] Salameh B. , Daibes A. G. , and Qaddumi J. , Assessing the Prevalence, Predictors, and Consequences of Secondary Traumatic Stress Among Emergency Nurses in Palestine During the COVID-19 Pandemic, SAGE Open Nursing. (2023) 9, 10.1177/23779608231207224. PMC10566272 37830081

[38] Lee M. H. , Applying Confucian Principles to the Resolution of Clinical Ethical Dilemmas in Taiwan, Journal of Nursing. (2018) 65, no. 6, 95–103, 10.6224/JN.201812_65(6).12, 2-s2.0-85057520872. 30488417

[39] Acoba E. F. , Social Support and Mental Health: The Mediating Role of Perceived Stress, Frontiers in Psychology. (2024) 15, 10.3389/fpsyg.2024.1330720. PMC10915202 38449744

[40] Schubert S. J. and Ringeisen T. , Mayer C.-H. and Vanderheiden E. , Coping and Stress-Related Emotions From a Cross-Cultural Perspective, International Handbook of Emotions: Resourceful Cultural Perspectives, 2025, Springer, Nature Switzerland AG, 61–74, 10.1007/978-3-031-86449-0_5.

[41] Mashego B. B. , Boshoff P. J. , and Fourie E. , Association Between Coping Strategies and Secondary Traumatic Stress Among Forensic Social Workers in South Africa, Social Work/Maatskaplike Werk. (2023) 59, no. 4, 378–402, 10.15270/59-4-1175.

[42] Vukčević Marković M. and Živanović M. , Coping With Secondary Traumatic Stress, International Journal of Environmental Research and Public Health. (2022) 19, no. 19, 10.3390/ijerph191912881. PMC9564895 36232179

[43] Cook R. M. and Fye H. J. , Trauma-Informed Supervision and Related Predictors of Burnout and Secondary Traumatic Stress Among Prelicensed Counsellors During the COVID-19 Pandemic, International Journal for the Advancement of Counselling. (2023) 45, no. 2, 310–329, 10.1007/s10447-022-09493-x. PMC9589689 36312764

[44] Chen S. C. and Chen C. F. , Antecedents and Consequences of Nurses’ Burnout: Leadership Effectiveness and Emotional Intelligence as Moderators, Management Decision. (2018) 56, no. 4, 777–792, 10.1108/MD-10-2016-0694, 2-s2.0-85044060162.

[45] Chew Q. H. , Wei K. C. , Vasoo S. , Chua H. C. , and Sim K. , Narrative Synthesis of Psychological and Coping Responses Towards Emerging Infectious Disease Outbreaks in the General Population: Practical Considerations for the COVID-19 Pandemic, Singapore Medical Journal. (2020) 61, no. 7, 350–356, 10.11622/smedj.2020046. 32241071 PMC7926608

[46] Morina N. , Weilenmann S. , Dawson K. S. et al., Efficacy of a Brief Psychological Intervention to Reduce Distress in Healthcare Workers During the COVID-19 Pandemic: A Randomized Controlled Trial, Psychological Trauma. (2023) 15, no. Suppl 2, S371–S383, 10.1037/tra0001524. 38885428

[47] Ogińska-Bulik N. and Michalska P. , Psychological Resilience and Secondary Traumatic Stress in Nurses Working With Terminally Ill patients—The Mediating Role of Job Burnout, Psychological Services. (2021) 18, no. 3, 398–405, 10.1037/ser0000421. 32091233

[48] Ramachandran H. J. , Bin Mahmud M. S. , Rajendran P. , Jiang Y. , Cheng L. , and Wang W. , Effectiveness of Mindfulness‐Based Interventions on Psychological Well‐Being, Burnout and Post‐Traumatic Stress Disorder Among Nurses: A Systematic Review and Meta‐Analysis, Journal of Clinical Nursing. (2023) 32, no. 11-12, 2323–2338, 10.1111/jocn.16265. 35187740

